# Periplasmic chaperone FkpA is essential for imported colicin M toxicity

**DOI:** 10.1111/j.1365-2958.2008.06327.x

**Published:** 2008-07-09

**Authors:** Julia Hullmann, Silke I Patzer, Christin Römer, Klaus Hantke, Volkmar Braun

**Affiliations:** 1 Microbiology/Membrane Physiology, University of TübingenAuf der Morgenstelle 28, D-72076 Tübingen, Germany; 2 Max Planck Institute for Developmental Biology, Department of Protein EvolutionSpemannstr. 35, 72076 Tubingen, Germany

## Abstract

Chaperones facilitate correct folding of newly synthesized proteins. We show here that the periplasmic FkpA chaperone is required for killing *Escherichia coli* by colicin M entering cells from the outside. Highly active colicin M preparations were inactive against *fkpA* mutant cells; 10^4^-fold dilutions killed *fkpA*^+^ cells. Three previously isolated spontaneous mutants tolerant to colicin M carried a stop codon or an IS*1* insertion in the peptidyl-prolyl-*cis-trans*-isomerase (PPIase) domain (C-domain) of FkpA, which resulted in deletion of the domain. A randomly generated mutant carried a G148D mutation in the C-domain. A temperature-sensitive mutant tolerant to colicin M carried a Y25N mutation in the FkpA N-domain. Mutants transformed with wild-type *fkpA* were colicin M-sensitive. Isolated FkpA-His reduced colicin M-His cleavage by proteinase K and renatured denatured colicin M-His *in vitro*; renaturation was prevented by the PPIase inhibitor FK506. In both assays, periplasmic SurA-His had no effect. No other tested periplasmic chaperone could activate colicin M. Among the tested colicins, only colicin M required FkpA for activity. Colicin M bound to cells via FhuA was inactivated by trypsin; unbound colicin M retained activity. We propose that colicin M unfolds during import across the outer membrane, FkpA specifically assists in folding colicin M into an active toxin in the periplasm and PPIase is essential for colicin M activity. Colicin M is a suitable tool for the isolation of FkpA mutants used to elucidate the functions of the FkpA N- and C-domains.

## Introduction

Colicins are toxic proteins produced by *Escherichia coli* that kill sensitive *E. coli* cells (Cascales *et al*., 2007). Colicin M is synthesized by *E. coli* cells carrying a ColBM plasmid. This colicin lyses sensitive cells by interfering with murein (peptidoglycan) biosynthesis (Braun *et al*., 1974; Schaller *et al*., 1982). In the absence of colicin M, the lipid-linked precursor of murein biosynthesis, undecaprenyl-*N*-acetyl-muramyl pentapeptide-*N*-acetyl-glucosamine (lipid II), is transferred across the cytoplasmic membrane and incorporated into murein. Undecaprenyl pyrophosphate is released and converted to the monophosphate, which re-enters the reaction cycle. Colicin M inhibits this undecaprenylphosphate (lipid) carrier regeneration step of murein synthesis (Harkness and Braun, 1989). This finding was recently confirmed and the exact target of colicin M was identified; colicin M is a phosphatase that cleaves the phosphate bond between the lipid moiety and the pyrophosphoryl group of lipid II (El Ghachi *et al*., 2006), forming 1-pyrophospho-*N*-acetyl-muramyl pentapeptide-*N*-acetyl-glucosamine and undecaprenol instead of undecaprenol pyrophosphate; undecaprenol does not enter the murein biosynthesis cycle.

Colicin M, like other colicins, is only bactericidal when provided from outside cells (Harkness and Braun, 1990) because it has access to the target only while entering the cells. Cells synthesizing colicin M are protected by an immunity protein that inactivates colicin M in the periplasm before it reaches its target in the cytoplasmic membrane (Ölschläger *et al*., 1991; Groß and Braun, 1996).

Uptake of colicin M into sensitive cells requires energy provided by the proton motive force across the cytoplasmic membrane (Braun *et al*., 2002). Uptake requires the FhuA outer membrane receptor and the TonB, ExbB and ExbD proteins, which form an energy-coupling device between the cytoplasmic membrane and the outer membrane (Braun, 1995; Postle and Kadner, 2003). Mutations in any of these genes render cells resistant to colicin M. We previously isolated mutants of *E. coli tol*erant to colicin *M* (*tolM*). These mutants are insensitive to colicin M, but the mutations map close to the streptomycin-resistance gene *rpsL* and not among the genes required for uptake (Braun *et al*., 1980). We have also isolated a temperature-sensitive *E. coli* mutant tolerant to colicin M at 42°C but sensitive at 30°C. This mutation also maps close to *rpsL* (Schaller *et al*., 1981), but we were unable to identify the mutated gene(s).

In a current study aimed at defining the role of periplasmic chaperones in the assembly of the FhuA outer membrane protein, we discovered that mutants in the *fkpA* gene, which encodes a periplasmic chaperone, are specifically resistant to high colicin M concentrations. No other tested chaperone mutant conferred colicin M resistance. As *fkpA* maps close to *rpsL*, *tolM* might be identical to *fkpA*.

**Fig. 1:**
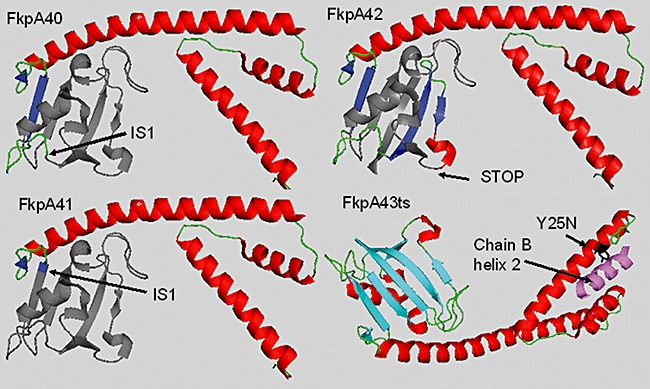
Location of mutations in FkpA. The mutations described here are indicated in the crystal structure model of FkpA (Saul *et al*., 2004). FkpA40 (from mutant Mo3) contains an IS*1* insertion close to the C-terminal end of the N-domain (chaperone domain), FkpA41 (from mutant Mo4) contains an IS*1* insertion in the bridge that links the N-domain to the C-domain (PPIase domain), FkpA42 (from mutant Mo6) contains a stop codon in the C-domain and the temperature-sensitive FkpA43 (from mutant K458) carries a Y25N exchange in the N-domain. This residue is involved in binding of helix 2 of the second FkpA molecule in the FkpA dimer. The views of the N-domain (helices) and C-domain (β-structure) differ to show the location of the mutations. The grey portions indicate deleted segments.

Periplasmic chaperones assist in the assembly of outer membrane proteins (Mogensen and Otzen, 2005; Betton, 2007). They also prevent aggregation of misfolded periplasmic protein derivatives (Arié *et al*., 2001; Ramm and Plückthun, 2001; Hu *et al*., 2006). FkpA consists of a mainly α-helical N-domain with a predicted chaperone function and an anti-parallel β-pleated sheet C-domain with peptidyl-prolyl-*cis-trans*-isomerase (PPIase) activity (Fig. 1). FkpA is inhibited by the immunosuppressant FK506 (Saul *et al*., 2004). FkpA and other chaperones are synthesized in response to extracytoplasmic stress (Sklar *et al*., 2007). FkpA and other periplasmic chaperones have been to date only implicated in assisting folding and refolding, and preventing misfolding of newly synthesized exported proteins and have not been related to the activation of an imported protein. Here we show that *tolM* is identical to *fkpA* and that FkpA is essential for the activity of imported colicin M.

## Results

### *fkpA* mutants are insensitive to colicin M

FhuA is a multifunctional protein that serves as receptor for colicin M and the phages T1, T5 and ϕ80 and as transporter for ferrichrome and the structurally related antibiotic albomycin (Braun *et al*., 2004). We have studied the role of periplasmic chaperones in the incorporation of complete FhuA and separately synthesized FhuA cork and FhuA barrel into the outer membrane of *E. coli* (Braun *et al*., 2003). Incorporation of active FhuA protein into the outer membrane of *E. coli* is determined by measuring the receptor and transport activities.

One mutant with a deleted periplasmic chaperone gene, *E. coli* JW3309 Δ*fkpA*, was insensitive to an undiluted colicin M sample; when this sample was diluted up to 10^4^, it inhibited growth of the BW25113 parent strain (Table 1). Complementation of strain JW3309 with wild-type *fkpA* cloned in plasmid pYH17 fully restored sensitivity to colicin M (Table 2). Strains mutated in the periplasmic chaperones Skp, SurA, PpiD or PpiA were as sensitive to colicin M as the wild-type strain (Table S1; the *skp* mutant is not listed, but was as sensitive as the parent strain, i.e. to a 10^4^ dilution).

**Table 1:** Sensitivity of *E. coli* strains to FhuA ligands.

Strain	Albomycin	Colicin M	T5	ϕ80
BW25113 *fkpA* wild-type	5	4	5	5
JW3309 Δ*fkpA*	5	i	5	5
AB2847 *fkpA* wild-type	5	4	5	5
Mo3 *fkpA40*	5	i	5	4
Mo4 *fkpA41*	5	i	4	5
Mo6 *fkpA42*	5	i	5	5
K458 *fkpA43*ts^a^	5	4	4	5
AB2847^b^	5	4	4	2
K458 *fkpA43*ts^b^	5	(3)	4	2
H5859 *ompT fkpA40*	5	i	4	5

a Cells were grown at 30°C.

b Cells were grown at 42°C.

The *E. coli* BW25133 and AB2847 are *fkpA*^+^ strains. The stock solutions of the ligands were diluted as described in the *Experimental procedures*, spotted onto nutrient agar plates seeded with the strains listed, and incubated; the zones of growth inhibition were determined. The numbers indicate the highest dilution that caused a clear zone of growth inhibition. i, insensitive; number in parentheses, very turbid inhibition zone.

**Table 2:** Complementation of *fkpA* mutants.

Strain	Temperature (°C)	Colicin M
JW3309 FkpA^a^	42	5
	27	4 (5)
JW3309 FkpA43-His	42	(2)
	27	3 (4)
JW3309 FkpA42-His	37	i^b^
Mo3 FkpA	42	5
	27	3 (5)
Mo3 FkpA-His^c^	42	5
K458 FkpA	42	5
	27	4 (5)
K458 FkpA-His	42	4 (5)
	27	4
K458 FkpA43-His	42	i
	27	4

a FkpA indicates that cells were transformed with pYH17 encoding FkpA.

b Insensitive.

c FkpA-His indicates that cells were transformed with pYH15 encoding FkpA-His. FkpA43-His indicates that cells were transformed with pH 16 encoding the temperature-sensitive FkpA43-His.

The sensitivity of the Δ*fkpA* mutant to the other FhuA ligands was as high as the sensitivity of the FkpA^+^ parent strain BW25113 (Table 1), which indicates that the lack of FkpA did not affect FhuA receptor activity. As small differences in transport can be more easily observed than small differences in receptor activities, we compared the ferrichrome transport rate of the Δ*fkpA* mutant with that of the wild-type strain. The Δ*fkpA* mutant and the wild-type strain had the same ferrichrome transport rate. The Δ*fhuA* mutant did not transport ferrichrome (Fig. S1).

The insensitivity of the Δ*fkpA* mutant to colicin M was not caused by structural changes in any of the proteins involved in colicin M uptake as transport of ferrichrome and sensitivity to albomycin and ϕ80 require the same TonB, ExbB and ExbD proteins as sensitivity to colicin M.

### *tolM* mutants are mutated in fkpA

In 1980/81, we isolated various *E. coli* mutants (strains Mo3, Mo4, Mo6 and K458) insensitive to colicin M but fully sensitive to albomycin and phages T1 and T5 (Braun *et al*., 1980). Mutant K458 is sensitive to colicin M at 30°C but insensitive at 42°C (Schaller *et al*., 1981). The *tolM* mutations map close to the *rpsL* locus. At the time of their isolation, no gene in this region was known to be specifically involved in colicin M sensitivity. The finding that *fkpA* mutants are insensitive to colicin M and map close to *rpsL* prompted us to sequence the previously isolated *tolM* mutant genes. Mutants Mo3 and Mo4 contain an IS*1* insertion in *fkpA* after nucleotide 498 and 457, respectively, of the coding sequence, resulting in protein fragments of 167 and 160 residues (including some residues of the translated IS*1* DNA; wild-type FkpA contains 245 residues). The truncated FkpA derivatives comprise the entire FkpA N-domain [residues 5–114 (Saul *et al*., 2004)]. In mutant Mo6, codon 164, GAA, was converted to the stop codon TAA. The temperature-sensitive mutant K458 contains a Y25N mutation in the N-domain (Fig. 1).

To further relate colicin M insensitivity to the lack of FkpA, the *tolM* (*fkpA*) mutants were complemented with wild-type FkpA, and sensitivity to colicin M was examined at 27°C and 42°C. Mutant Mo3 regained sensitivity, but was less sensitive at 27°C than at 42°C (Table 2), which presumably reflects a lower uptake rate and lipid II cleavage rate at 27°C. The FkpA-complemented temperature-sensitive mutant K458 was sensitive at 27°C and 42°C. When mutant K458 was complemented with the temperature-sensitive FkpA (FkpA43-His) encoded on pYH16, cells were sensitive at 27°C and insensitive at 42°C. Strain JW3309 *fkpA* transformed with wild-type *fkpA* became sensitive, displayed temperature sensitivity when transformed with *fkpA43* and remained insensitive when transformed with *fkpA42*. These data clearly relate colicin M sensitivity to FkpA.

### FkpA restores activity of denatured colicin M

**Fig. 2:**
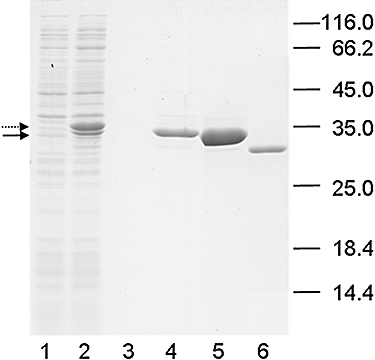
SDS-PAGE of purified FkpA-His (lane 5) and purified colicin M (lane 6). FkpA from *E. coli* BL21(DE3) pYH15 *fkpA* was affinity-purified on a Ni-NTA agarose column. FkpA isolated from cells (lanes 1 and 2) and culture supernatants (lanes 3 and 4), prior to (lanes 1 and 3) and after IPTG induction (lanes 2 and 4) were subjected to SDS-PAGE. The FkpA precursor is indicated by a dotted line, processed FkpA by a solid line. The positions and molecular masses (kDa) of the standard proteins are indicated on the right.

To examine whether FkpA affects the structure of colicin M, the proteins were purified from *E. coli* BL21(DE3) transformed with pYH15, which carries *fkpA* with an encoded C-terminal His tag, and from *E. coli* BL21 *fhuA* transformed with pMLD237, which carries *cma* with an encoded N-terminal His tag. The *fhuA* mutant lacks the colicin M receptor and is therefore not killed by colicin M synthesized in the absence of the colicin M immunity protein (Harkness and Braun, 1990). The *fkpA* and *cma* genes were transcribed by the phage T7 RNA polymerase under *lacUV5* control. After IPTG induction, three- to fivefold more FkpA was in the culture supernatant than in the cell pellet, owing to cell lysis (90%). In contrast, more than 90% of colicin M was cell-associated. FkpA-His and colicin M-His were purified by affinity chromatography on Ni-NTA agarose. No proteins other than FkpA-His and colicin M-His were observed after SDS-PAGE Fig. 2).

The *in vitro* PPIase activity of purified FkpA-His (Fig. 2, lane 5) was tested with an established refolding assay using RNase T1 denatured in 5.6 M guanidine hydrochloride (Ramm and Plückthun, 2000). RNase refolding was monitored spectroscopically as the increase in fluorescence over time. FkpA-His (35 nM) increased RNase T1 refolding 27-fold over spontaneous refolding, and 10 nM FkpA-His increased refolding eightfold. The immunosuppressant FK506 (12 μM) completely inhibited RNase T1 refolding by 35 nM FkpA.

The same FkpA-His sample used for RNase T1 refolding was used to refold colicin M-His denatured in 5 M guanidine hydrochloride. With the denaturing conditions used (10°C overnight), a residual colicin M activity of 2–5% remained. Incubation with FkpA-His for 2 h at 37°C increased colicin M activity 10-fold to 25–50% of the original activity of the native sample (Table 3). Addition of FK506 completely inhibited the increase of colicin M activity by FkpA-His. The purified FkpA313-His protein, which has a G148D mutation in the PPIase domain, did not activate denatured colicin M. Purified SurA-His and lysozyme had no effect. These results indicate a specific activation of colicin M by FkpA and that PPIase activity is essential for activation. Attempts to spectroscopically monitor colicin M denaturation and renaturation yielded no suitable wavelength at which fluorescence changes reflected structural changes.

**Table 3:** Reactivation of denatured colicin M by FkpA.

Proteins	Colicin M activity (%)
Native colicin M-His	100
Native colicin M-His + FkpA-His	100
Denatured colicin M-His	2–5
Denatured colicin M-His + FkpA-His	25–50
Denatured colicin M-His + FkpA-His + ethanol	25–50
Denatured colicin M-His + FkpA-His + FK506	2–5
Denatured colicin M-His + FkpA313-His	3–4
Denatured colicin M-His + SurA-His	3–4
Denatured colicin M-His + lysozyme	3–4

Colicin M-His was denatured in 5 M guanidine hydrochloride at 10°C.

### FkpA reduces inactivation of colicin M by proteinase K

To further test interaction of colicin M with FkpA, we examined whether FkpA-His prevents degradation of colicin M-His by proteinase K. Colicin M-His was inactivated by proteinase K through degradation, and FkpA-His partially prevented the degradation of colicin M-His (Table 4). Protection of colicin M-His by active FkpA-His was specific as the inactive FkpA313-His mutant and SurA-His did not inhibit colicin M-His inactivation.

**Table 4:** FkpA reduces inactivation of colicin M by proteinase K.

	Diameter of lysis zones (mm) Colicin M-His dilution			
	
Sample	10°	10^1^	10^2^	10^3^
Colicin M-His	0	0	0	0
Colicin M-His and FkpA-His	7.5	5	0	0
Colicin M-His and FkpA313-His	0	0	0	0
Colicin M-His and SurA-His	0	0	0	0
Colicin M-His, no proteinase K	10.5	8	5.5	0

All samples were treated with proteinase K unless indicated otherwise. Colicin M-His samples were applied to a nutrient agar plate seeded with the sensitive *E. coli* AB2847 strain. After incubation overnight, the diameter of the zones of growth inhibition was measured.

### FkpA does not activate newly synthesized colicin M

FkpA might not only be involved in activation of imported colicin M, but possibly also be involved in activation of exported colicin M. To test this possibility, we isolated colicin M from strains H5859 *fkpA40* and BL21 *fhuA fkpA*^+^, both of which had been transformed with pMLD237, which carries a *cma* gene encoding colicin M with a C-terminal His tag. Crude cell extracts from both strains and colicin M-His purified on a Ni-NTA agarose column were equally active in killing cells of strain AB2847 on nutrient agar plates (10^4^ dilution), which showed that FkpA does not activate newly synthesized colicin M during export.

As most of the overexpressed FkpA was found in the culture medium, we examined whether chromosomally encoded FkpA can be found in the spent medium of cells grown without apparent stress. Concentrated spent medium contained FkpA, as evidenced by a corresponding band on SDS-PAGE, and RNase T1 refolding activity was found. It was therefore possible that secreted FkpA changes the structure of colicin M to an import-compatible conformation. However, addition of purified FkpA to colicin M synthesized by *fkpA*^+^ and *fkpA40* mutant cells did not result in killing of the *fkpA*40 mutant.

**Fig. 3:**
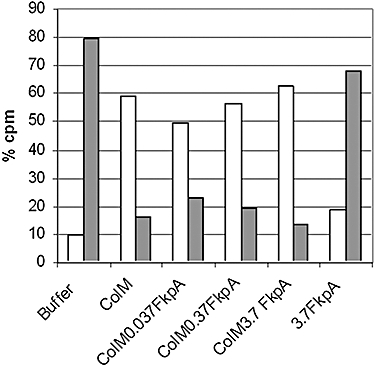
Release of undecaprenol from lipid II by colicin M. [^14^C] *N*-Acetyl-glucosamine-labelled lipid II was incubated with 0.3 μg of colicin M (ColM) and different concentrations of FkpA as indicated (values given in μg). The reaction products (PP-muropeptide, white bars; lipid II, grey bars) were separated by thin-layer chromatography, and the products were scraped off the thin-layer plates at the positions identified with iodine vapour and counted in a liquid scintillation counter.

We incubated colicin M purified from *fkpA*^+^ and *fkpA* mutant cells with lipid II and determined the release of undecaprenol by thin-layer chromatography. Colicin M isolated from *E. coli* H5859 *fkpA40* and from *E. coli* BL21 *fhuA fkpA*^+^ (not shown) cleaved approximately 50% of the added lipid II (Fig. 3). These results indicated that FkpA does not activate newly synthesized colicin M.

We also tested whether isolated colicin M could be activated by isolated FkpA. Lipid II was incubated with colicin M isolated from *fkpA40* cells and increasing concentrations of FkpA. Colicin M (0.6 μg) cleaved 50–60% of the substrate (Fig. 3) and was only slightly activated by added FkpA. These results support the finding that active colicin M is synthesized in *fkpA*^+^ and *fkpA* cells.

### Colicin M changes its structure when bound to cells

It is likely that colicin M is unfolded during import across the outer membrane into the periplasm. To examine whether colicin M changes its conformation when it binds to the FhuA outer membrane receptor, we tested its trypsin sensitivity. This approach was guided by our earlier finding that unpurified colicin M is resistant to degradation by trypsin but is trypsin-sensitive when bound to susceptible *E. coli* cells (Schaller *et al*., 1981). Purified colicin M (0.1 μg ml^−1^) was added to exponentially growing cultures of the wild-type *fhuA* strain AB2847 and the Δ*fhuA* strain MB97 to exclude unspecific cell adsorption of colicin M as the cause of trypsin sensitivity. Cultures were treated with trypsin, and the remaining colicin M activity in the culture supernatant was determined in a plate assay with a colicin M-sensitive strain. A surplus of trypsin inhibitor was added prior to spotting the spent medium onto plates seeded with the colicin M-sensitive strain to avoid trypsin action while colicin M entered the indicator bacteria. The control medium contained colicin M but no cells. No colicin M activity was found in the supernatant of the *fhuA*^+^ strain, only a slight reduction in colicin M activity was observed in the supernatant of the Δ*fhuA* strain, and no reduction in colicin M activity was found in the control (Table 5), i.e. colicin M was degraded by trypsin only in cultures of *fhuA*^+^ cells.

**Table 5:** Degradation of cell-bound colicin M by trypsin.

Strain	Col M-His	Col M-His + trypsin + inhibitor
AB2847 *fhuA*^+^	9	0
MB97 *fhuA*	9	8
Uninoculated medium	9	9

The numbers indicate the diameter of lysis zones in mm obtained by spotting 10 μl of spent medium onto a nutrient agar plate seeded with the colicin M (Col M)-sensitive strain *E. coli* AB2847.

**Fig. 4:**
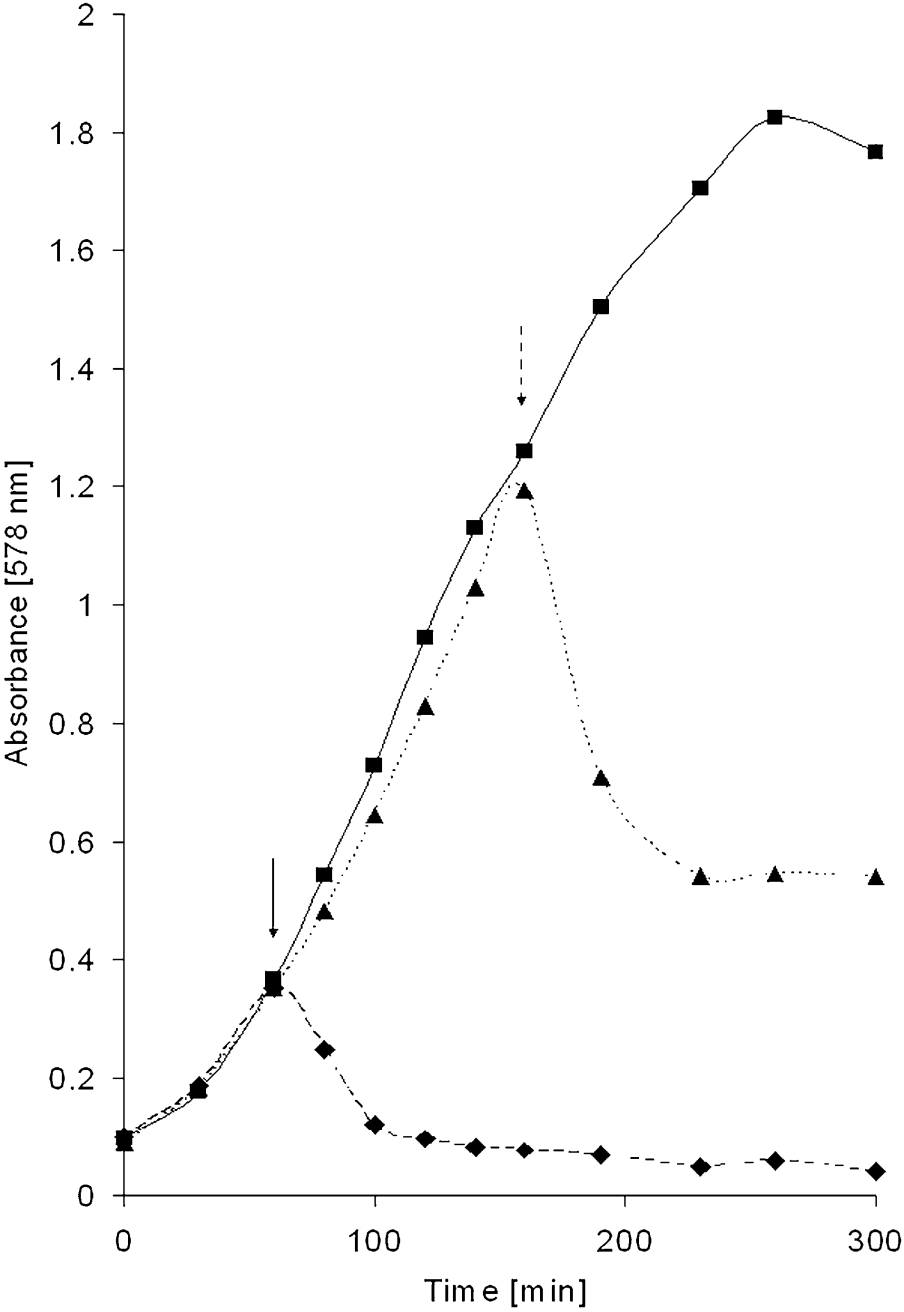
Growth of *E. coli* AB2847 in the presence of colicin M and colicin M with trypsin. Cells were grown in LB medium, and the absorbance at 578 nm was monitored. (♦) Colicin M (1 μg ml^−1^) added after 60 min incubation (solid arrow); (▴) colicin M (1 μg ml^−1^) and trypsin (0.3 mg ml^−1^) added together after 60 min incubation, and trypsin inhibitor (1.5 mg ml^−1^) added after 160 min incubation (dashed arrow); (▪) trypsin (0.3 mg ml^−1^) added after 60 min incubation (control).

We monitored cell growth in the presence of colicin M and colicin M with trypsin in another experiment. A surplus of colicin M (1 μg ml^−1^) was used to monitor the action of colicin M after addition of trypsin inhibitor. Trypsin prevented cell lysis by colicin M (Fig. 4). Addition of trypsin inhibitor resulted in the lysis of cells by colicin M that had not been degraded by trypsin (Fig. 4). When colicin M was completely inactivated by trypsin (initial colicin M concentration of 0.1 μg ml^−1^; Table 5), addition of trypsin inhibitor did not result in cell lysis (not shown), which supports the conclusion that colicin M and not FhuA was degraded by trypsin. In the presence of trypsin inhibitor, synthesis of FhuA would render cells susceptible to colicin M killing. We and others have previously shown that FhuA in cells and isolated outer membranes is not degraded by trypsin (Hoffmann *et al*., 1986; Moeck *et al*., 1996; Bonhivers *et al*., 2001).

### Temperature-sensitive FkpA shows an increased sensitivity to proteinase K

**Fig. 5:**
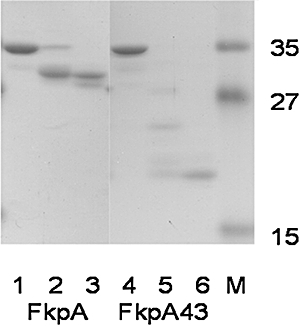
Susceptibility of wild-type FkpA-His (lanes 1–3) and the temperature-sensitive FkpA43-His (lanes 4–6) to proteinase K. The isolated proteins were incubated at 4°C with proteinase K (lanes 2 and 5, 1.5 ng; lanes 3 and 6, 6.5 ng). M, molecular mass standards in kDa.

To examine whether temperature-sensitive FkpA43 assumes a structure that differs from wild-type FkpA, isolated FkpA43 was incubated with proteinase K at 4°C. The low temperature was used to delay cleavage so that the larger fragments formed could be identified by SDS-PAGE. FkpA43 was cleaved by proteinase K to smaller fragments than those formed by cleavage of wild-type FkpA (Fig. 5), which indicates that FkpA43 and wild-type FkpA have different conformations. Although FkpA-43 was active at 27°C, its altered structure was clearly discernible at 4°C.

### Chaperone/PPIase dependence is unique to colicin M toxicity

The previously isolated *tolM* mutants were sensitive to colicins B, Ib and E1, which are taken up through receptors other than FhuA (Braun *et al*., 1980). Like colicin M, colicins B and Ib also require TonB, ExbB and ExbD (Ton system) for uptake; colicin E1 requires TolA, TolQ and TolR (Tol system). In the present study, we tested additional colicins (A, D, E3, K, L, N, S4, U, 5 and 10) that are taken up by either the Ton system or the Tol system, use various receptors and form pores in the cytoplasmic membrane or degrade RNA and DNA in the cytoplasm. *E. coli* JW3309 Δ*fkpA* was fully sensitive to these colicins (Table S1), which showed that the *fkpA* mutation only conferred colicin M resistance. Mutants carrying mutations in other chaperone genes and deletion mutants in *groL*, *secB*, *clpP* and *dnaK* were sensitive to all tested colicins (Table S1). Therefore, FkpA plays a unique role for colicin M activity.

## Discussion

In this study, we show for the first time that the activity of an imported protein, colicin M, depends specifically on a single periplasmic chaperone, FkpA. Four *fkpA* mutants were completely insensitive to colicin M, and a temperature-sensitive *fkpA* mutant was nearly insensitive at the non-permissive temperature.

The Y25N replacement in FkpA43 causing the temperature sensitivity is located in helix 1, which is part of the interface between the two FkpA subunits that form a dimer (Fig. 1) (Saul *et al*., 2004). It is likely that the amino acid replacement weakens the interaction between the two subunits so that inactive monomers are formed at 42°C. The altered structure of FkpA43 was evident even at 4°C, at which it was degraded by proteinase K to a greater extent than wild-type FkpA. The phenotype of the colicin-resistant *fkpA* mutants and also the restoration of colicin M sensitivity of the mutants by wild-type *fkpA* and the acquisition of the temperature-sensitive phenotype of *fkpA* mutants when transformed with *fkpA43ts* clearly relate colicin M sensitivity to FkpA. Conformational changes in colicin M elicited specifically by active FkpA was demonstrated by the partial restoration of activity of denatured colicin M by added FkpA and lack of restoration by inactive FkpA313, SurA and lysozyme. Moreover, only active FkpA reduces cleavage of colicin M by proteinase K. In this case, only active FkpA binds to colicin M, or the FkpA PPIase activity constantly shifts the equilibrium between a sensitive and resistant form of colicin M towards the resistant form.

FkpA is not required for the activity of newly synthesized colicin M as colicin M isolated from *fkpA* mutant cells was active *in vivo* and *in vitro*. FkpA is only required for cell lysis when added from the outside. Then how is colicin M inactivated during uptake and how does FkpA activate colicin M in the periplasm? It is unlikely that colicin M is synthesized in both a prolyl-*cis* and a prolyl-*trans* form and that only the *cis* form is taken up and activated by FkpA in the periplasm. In this case, addition of FkpA together with colicin M to cells would inactivate colicin M, which was not observed. The target of colicin M resides at the outer surface of the cytoplasmic membrane. The lipid carrier translocates the hydrophilic murein precursor to the periplasmic side of the membrane, where it is incorporated into murein. To reach its target, colicin M binds to FhuA in the outer membrane, which presumably transfers the colicin into the periplasm as energy coupling of FhuA to TonB is required for colicin M sensitivity. Mutants in the TonB box of FhuA are colicin M-resistant. However, we cannot exclude that binding of colicin M to FhuA requires TonB, as binding of phages T1 and ϕ80 to FhuA requires TonB (Schöffler and Braun, 1989). It is unknown whether the entire colicin M is taken up into the periplasm. The C-proximal activity domain entering the periplasm would suffice. Colicin M, like all studied colicins, is composed of three structural and functional domains: an N-terminal translocation domain, a central receptor binding domain and a C-terminal activity domain. A 24 kDa colicin M fragment that lacks 5 kDa from the N-terminus binds to the FhuA receptor but does not kill cells (Dreher *et al*., 1985). Point mutations that inactivate colicin M activity reside in the C-terminal domain (Pilsl *et al*., 1993). Regardless whether the entire colicin or only the activity domain is translocated across the outer membrane, it most likely unfolds partially and rearranges its functional domains.

The finding that colicin M becomes trypsin-sensitive upon binding to cells via FhuA suggests a conformational change of colicin M close to the cell surface, when it is still accessible to trypsin. For other colicins, there is evidence that colicin domains physically separate from each other during import but most of them remain chemically linked. For example, colicin E2 remains bound to the outer membrane receptor when its nuclease domain enters the cytoplasm (Sharma *et al*., 2007). In the crystal structures of the pore-forming colicin Ia (Wiener *et al*., 1997) and the nuclease E3 (Soelaiman *et al*., 2001), the receptor binding domains are separated over a large distance from the translocation and activity domains. The three domains must further separate during import so that the activity domains can insert into the cytoplasmic membrane and enter the cytoplasm. When colicin Ia is bound to its Cir receptor, the translocation and activity domains of colicin Ia extend ∼80 Å away from Cir and ∼150 Å from the lipid bilayer (Buchanan *et al*., 2007). From the structure of the colicin E2 binding domain bound to its BtuB receptor, it was inferred that the projection of the binding domain in the plane of the outer membrane is ∼70 Å and that of the entire colicin ∼100 Å (Sharma *et al*., 2007). The structural data imply that for translocation of the activity domain across the outer membrane and subsequent insertion into the cytoplasmic membrane or uptake into the cytoplasm, large movements of the translocation and activity domains are required, and these movements are most likely accompanied by substantial structural changes.

Experimental evidence for structural changes of the activity domain has been obtained with those colicins that are exported with immunity proteins, which are tightly bound to the activity domains. These colicins undergo structural changes in the activity domains to dissociate from the immunity proteins (Zakharov and Cramer, 2004; Cascales *et al*., 2007). Colicin A shows an increased activity after urea treatment (Benedetti *et al*., 1992) and disulphide bonds engineered into the pore-forming domain delay translocation (Duché *et al*., 1994). Similar structural changes are expected for colicin M during uptake into sensitive cells. Trypsin sensitivity of cell-bound colicin M and the requirement for FkpA for colicin M activity support a change in conformation. It is difficult to determine such a structural change of the imported colicin M in the periplasm as a few molecules (approximately 10) suffice to kill a cell (Schaller *et al*., 1981). Therefore, we denatured purified colicin M *in vitro* and determined restoration of colicin M activity by FkpA. Colicin M denatured in 5 M guanidine hydrochloride was activated by FkpA. Although the denatured colicin M most likely does not reflect the form of colicin M after translocation across the outer membrane, restoration of activity demonstrates interaction of FkpA with colicin M such that folding into a native conformation is enhanced. FkpA may cause refolding by catalysing proline *cis-trans* isomerization, and may accelerate spontaneous folding by binding to the correctly folded protein, which is withdrawn from the equilibrium with incorrectly folded protein forms. As FK508 inhibits refolding, it is likely that the PPIase activity of FkpA is required. Colicin M contains 15 proline residues (5.5%), which is higher than the average proline content (4.8%) of all protein sequences in the Swiss-Prot database (http://expasy.org/sprot/relnotes/relstat.html). One or several of the proline residues could serve as *cis-trans* isomerization substrates. However, refolding of colicin M does not necessarily involve proline *cis-trans* isomerization as FkpA increased solubility of an antibody fragment devoid of *cis*-prolines in the periplasm (Bothmann and Plückthun, 2000).

Our results indicate that colicin M can be used to isolate *fkpA* mutants for which there is no other selection procedure. Mutations in chaperones do not lead to a severe impairment of the cell physiology as chaperones can mutually replace each other functionally (Rizzitello *et al*., 2001). For example, the periplasmic chaperone Skp binds to the OmpA protein. *skp* mutants have fewer outer membrane proteins (Chen and Henning, 1996). *skp* mutants show no physiological phenotype, but *skp degP* double mutants do not grow at 37°C and they accumulate proteins in the periplasm (Schäfer *et al*., 1999). Single mutations in the periplasmic chaperone gene *surA* lead to lower levels of porins and unfolded monomeric LamB maltoporin in the periplasm and cause hypersensitivity to bile salts, detergents and hydrophobic antibiotics (Rouviere and Gross, 1996; Rizzitello *et al*., 2001; Betton, 2007). *surA degP* double mutations are lethal. Also mutations in *fkpA*, which encodes the periplasmic chaperone studied here, lead to no observable phenotype. FkpA suppresses the formation of inclusion bodies in the periplasm by the folding-defective maltose binding protein MalE31 (Arié *et al*., 2001) and assists in the formation of a functional antibody fragment linked to a filamentous phage (Bothmann and Plückthun, 2000). A *degP fkpA* double mutant stops growing 3 h after shifting from 30°C to 40°C (Arié *et al*., 2001). FkpA mutants and colicin M as the natural substrate would be instrumental for correlating FkpA structure to FkpA function. The results of the present study indicate that the PPIase activity is essential for colicin M activation. The PPIase inhibitor FK506 inhibits colicin M renaturation, the G148D mutant in the PPIase domain is inactive and the N-proximal chaperone fragments of 127, 140 and 163 residues in the *fkpA* mutants are not sufficient to confer colicin M sensitivity.

The other colicins tested in our study, unlike colicin M, do not require FkpA and the other periplasmic PPIases/chaperones. Pesticin, which acts as a muramidase in the periplasm (Vollmer *et al*., 1997), is also active in an *fkpA* mutant. Localization of the target site does not play a role for colicin M activation by FkpA. The pore-forming colicins assume their active conformation while they insert into the cytoplasmic membrane and folding of the cytoplasmic RNase and DNase colicins may be assisted by cytoplasmic chaperones.

Our data are consistent with the proposal that colicin M unfolds during import and FkpA assists refolding of colicin M in the periplasm to its active form. Active colicin M can then access its substrate in the outer leaflet of the cytoplasmic membrane and cleave the phosphate bond between undecaprenol and PP-*N*-acetyl-muramyl pentapeptide-*N*-acetyl-glucosamine.

## Experimental procedures

### Bacterial strains and plasmids

The bacterial strains and plasmids used and their sources are listed in Table 6. The previously isolated *tolM* mutants were obtained by screening for colonies that grew on nutrient agar plates containing colicin M (Braun *et al*., 1980). Those colonies that were specifically insensitive to colicin M but fully sensitive to the FhuA ligands albomycin and phages T1 and T5 were collected. Sensitivity to these ligands excluded mutations in *fhuA*, *tonB*, *exbB* and *exbD.* Sensitivity to colicins B and Ib supported the conclusion of functional *tonB*, *exbB* and *exbD* genes. Unaltered sensitivity to antibiotics for which the outer membrane forms a partial permeability barrier suggested that the outer membrane structure was not altered.

**Table 6:** *E. coli* K-12 strains and plasmids used in this study.

Strain/Plasmid	Genotype	Reference
*Strain*		
AB2847	*aroB thi tsx malT*	Schaller *et al*. (1982)
BL21(DE3)	F^-^*ompTgal dcm lon hsd*_*B*_ ( 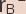 m_B_) λ(DE3) *lacI lacUV5*-T7 gene 1	Studier and Moffatt (1986)
BL21 *fhuA*	BL21(DE3) *fhuA*	This institute
BW25113	*lacI*^*q*^*rrnB3*Δ*lacZ4787 hsdR514*Δ(*araBAD*)*567*Δ(*rhaBAD*)*568 rph-1*	Keio Collection^a^
H5859	BL21(DE3) *rpsL fkpA40*	This study
JW0013	BW25113 Δ*dnaK*	Keio Collection^a^
JW0052	BW25113 Δ*surA*	Keio Collection^a^
JW0157	BW25113 Δ*degP*	Keio Collection^a^
JW0427	BW25113 Δ*clpP*	Keio Collection^a^
JW0431	BW25113 Δ*ppiD*	Keio Collection^a^
JW3309	BW25113 Δ*fkpA*	Keio Collection^a^
JW3326	BW25113 Δ*ppiA*	Keio Collection^a^
JW3584	BW25113 Δ*secB*	Keio Collection^a^
JW4103	BW25113 Δ*groL*	Keio Collection^a^
K458	AB2847 *fkpA43* (*tolM-*ts)	Schaller *et al*. (1981)
MB97	AB2847 Δ*fhuA*	Braun *et al*. (2003)
MC4100	F^-^Δ*lacU169 araD139 rpsL150 relA1 ptsF rbsflbB5301*	Casadaban (1976)
MC4100Skp	F^-^Δ*lacU169 araD139 rpsL150 relA1 ptsF rbsflbB5301 proAB::*Tn*10 Δskp*	Schäfer *et al.* (1999)
Mo3	AB2847 *fkpA40* (*tolM40*)	Braun *et al*. (1980)
Mo4	AB2847 *fkpA41* (*tolM41*)	Braun *et al*. (1980)
Mo6	AB2847 *fkpA42* (*tolM42*)	Braun *et al*. (1980)
SIP127	AB2847 *fkpA313*	This study
*Plasmid*		
pASKSurA	pASK75 encoding SurA-His	Behrens *et al*. (2001)
pMLD237	pET2430::*cma* encoding Cma-His	El Ghachi *et al*. 2006)
pSP127/56	pET25b encoding FkpA313-His^b^	This study
pYH15	pET25b encoding FkpA-His^b^	This study
pYH16	pET25b encoding FkpA43-His^b^	This study
pYH17	pT7-6 encoding FkpA	This study

a The strains of the Keio Collection, Japan, were described by Baba *et al*. (2006).

b FkpA-His denotes the FkpA protein with a C-terminal His tag.

Temperature-sensitive *tolM* mutants were screened on nutrient agar plates containing colicin M and ferrichrome as the sole iron source. Ferrichrome can be used as the iron source only when the *fhuA*, *tonB*, *exbB* and *exbD* genes are functional; use of ferrichrome therefore excludes mutations in these genes as the cause of colicin M insensitivity. Colonies that were colicin M-insensitive at 42°C but -sensitive at 30°C were collected.

The plasmid encoding colicin M modified at the N-terminus by a (His)_6_ tag was obtained from D. Mengin-Lecreulx (Université Paris-Sud, Orsay, France). Cells freshly transformed with the plasmids were used in the assays.

*fkpA40* and *rpsL* were co-transduced with phage P1 (selection for streptomycin resistance) from *E. coli* Mo3 into *E. coli* BL21(DE3) to yield strain H5859.

Primers FkpAxba (GCTCTAGAGTATGTAGATTTGTTCGACAACGC) and FkpAxho (GATCTCGAGTTTTTTAGCAGAATCTGCG) were both used to amplify the *fkpA* gene from *E. coli* AB2847. Plasmid pYH15 was generated by cloning the XbaI/XhoI-digested PCR product into plasmid pET25b; the recombinant FkpA contained a C-terminal His tag. Plasmid pYH16 was generated in the same way by cloning the PCR product of *E. coli* K458 into pET25b.

Plasmid pYH17 was generated by cloning the PCR fragment of the *fkpA* region of AB2847 into plasmid pT7-6 using the primers FkpAprom GTGAGATCTGTATGTAGATTTGTTCGACAACGC (BglII) and FkpAend GACCAATTGCACTCCTTTTCAGGAGCCTGTCG (MunI).

*fkpA313* was obtained by mutagenesis of *E. coli* AB2847 with *N*-methyl-*N*′-nitro-*N*-nitrosoguanidine (Miller, 1972) and selection for colicin M insensitivity and albomycin sensitivity (Braun *et al*., 1980). As albomycin requires the same genes for translocation across the outer membrane as colicin M, the procedure selects against colicin M uptake mutants. From the resulting strain SIP1275, *fkpA* chromosomal DNA was amplified by PCR with primers FkpA1 AGTATGTAGATTTGTTCGACAACGC and FkpA2 GTTTTTTAGCAGAATCTGCGGCTTTC. The product was cloned into pET25b cleaved with XhoI/XbaI and the overhanging ends were filled in. The resulting plasmid, pSP127/56, encodes FkpA313-His with the amino acid replacement G→D at position 148 of processed FkpA.

### Protein isolation

FkpA-His was isolated from cells of *E. coli* BL21(DE3) *fkpA* transformed with pYH15 encoding FkpA-His. Cells were grown with shaking at 37°C in Luria–Bertani (LB) medium (tryptone 10 g l^−1^, yeast extract 5 g l^−1^, NaCl 5 g l^−1^) with 50 μg ml^−1^ ampicillin to an OD of 0.5 at 600 nm; 1 mM IPTG was then added. After 3 h incubation, the culture was centrifuged, 50 ml of the clear supernatant was diluted 1:2 with buffer A (50 mM Na-phosphate buffer, 300 mM NaCl, 10 mM imidazole, pH 8.0), and the sample was applied to a Ni-NTA agarose column (1.5 ml bed volume) (Qiagen, Hilden, Germany) equilibrated with buffer A and washed with 10 ml of 50 mM Na-phosphate buffer, 300 mM NaCl, 20 mM imidazole, pH 8.0. The His-tagged proteins were eluted with 50 mM Na-phosphate buffer, 300 mM NaCl, 200 mM imidazole, pH 8.0. The fractions obtained were analysed by SDS-PAGE for their content of the desired proteins and impurities.

FkpA313-His was isolated accordingly from the FkpA313-His-encoding plasmid pSP127/56 to an electrophoretically homogeneous form.

SurA-His was isolated from MC4100 pASKSurA as described (Behrens *et al*., 2001).

(His)_6_-colicin M was synthesized in *E. coli* BL21 *fhuA* and purified on a Ni-NTA-agarose column as described (El Ghachi *et al*., 2006).

### Sensitivity tests

Sensitivity to colicin M and albomycin and to the phages T5 and ϕ80 was tested. Aliquots (7 μl) of 10-fold dilutions of colicin M (0.25 mg ml^−1^ stock solution), phage T5 and phage ϕ80 stock solutions, and a threefold dilution of an albomycin stock solution were spotted onto LB (10 g bacto tryptone, 5 g yeast extract, 5 g NaCl per l) agar plates seeded with the strains to be tested, as described previously (Braun *et al*., 1974). The results are given as the last 10-fold dilution that resulted in a clear zone of growth inhibition. For example, a colicin M titer of 4 means that a 10^4^-diluted stock solution inhibited cell growth. By plotting colicin M concentrations against the diameters of growth inhibition, a linear standard curve was obtained, which was used to estimate the degree of colicin M refolding by FkpA.

### Degradation of colicin M by trypsin

Susceptibility of purified colicin M to degradation by trypsin was tested with a plate assay and in liquid culture. In the plate assay, colicin M-His (0.1 μg ml^−1^) dissolved in the elution buffer used for Ni-NTA chromatography, supplemented with 0.1% dodecylmaltoside, was incubated in LB medium for 20 min with 0.3 mg ml^−1^ trypsin at 30°C; 1.5 mg ml^−1^ trypsin inhibitor was then added. Samples contained no cells, *E. coli* AB2847 or its *fhuA* deletion derivative MB97. The cultures were centrifuged, and colicin M activities were determined in the supernatant by spotting 10 μl onto plates seeded with *E. coli* AB2847. The diameter of the lysis zones was measured. In the liquid assay, colicin M-His (1 μg ml^−1^) was added after 60 min incubation to exponentially growing cells of *E. coli* AB2847 in LB medium. A second culture received 0.3 mg ml^−1^ trypsin together with colicin M-His. A third culture received colicin M and trypsin and then 1.5 mg ml^−1^ trypsin inhibitor after 160 min incubation. Growth of the cultures was monitored at 578 nm for 5 h.

### Reactivation of colicin M by FkpA

Colicin M (0.4 mg ml^−1^) was incubated overnight in 0.1 M Tris-HCl, pH 8, 5 M guanidine hydrochloride at 10°C. The solution was then diluted 100-fold by adding 50 mM Tris-HCl, pH 8, 50 mM NaCl, 5 mM dithiothreitol and incubated for 2 h at 37°C without addition or after addition of 17 μg ml^−1^ isolated FkpA-His; FkpA-His and 100 μM FK506 dissolved in ethanol (1% final concentration in the assay); 13 μg ml^−1^ isolated FkpA313-His; 18 μg ml^−1^ isolated SurA-His; 20 μg ml^−1^ hen egg white lysozyme; or 1% ethanol. Colicin M activity was determined by spotting 3 μl on a nutrient agar plate seeded with the indicator strain *E. coli* AB2847. The diameter of the zones of growth inhibition was measured and compared with a standard curve.

### Reduction in proteinase K cleavage of colicin M by FkpA

Colicin M-His (37 μg ml^−1^) was incubated in 0.1 M Tris-HCl, 50 mM NaCl, 5 mM CaCl_2_, 5 mM dithiothreitol with 0.25 mg ml^−1^ proteinase K for 13 min on ice. The reaction was stopped by adding 5 mM phenylmethanesulfonylfluoride. Additional assays contained 22 μg ml^−1^ FkpA-His, 13 μg ml^−1^ FkpA313-His or 18 μg ml^−1^ SurA-His and were pre-incubated for 30 min at 37°C. Colicin M activity was determined by spotting undiluted and 10-fold-diluted samples onto nutrient agar plates seeded with *E. coli* AB2847.

### Determination of PPIase activity *in vitro*

The *in vitro* PPIase activity of FkpA was determined using an RNase T1 refolding assay (Ramm and Plückthun, 2001). RNase T1 (Sigma) was unfolded by overnight incubation in 5.6 M guanidine hydrochloride in 0.1 M Tris-HCl pH 8 at 10°C. Refolding was initiated by 80-fold dilution to a final concentration of 0.2 μM in 50 mM Tris-HCl, pH 8, 50 mM NaCl and 10 nM FkpA-His (dimer) purified on a Ni-NTA agarose column (Fig. 2, lane 5). The folding reaction was followed at 10°C with a Jasco FP-6500 spectrofluorometer at 323 nm after excitation at 295 nm. Inhibition of the PPIase activity by FK506 was determined with 10 μM FK506 (final concentration) dissolved in ethanol. Controls contained denatured RNase T1 with FkpA, with and without ethanol, or only denatured RNase T1.

### Cleavage of lipid II by colicin M

Cleavage of radiolabelled lipid II by colicin M was determined essentially as described in El Ghachi *et al*. (2006). Purified colicin M (0.6 μg in 16 μl 0.1 M Tris-HCl, 20 mM MgCl_2_, 150 mM NaCl, pH 7.5) isolated from *E. coli* BL21(DE3) carrying wild-type *fkpA* or mutant *fkpA40* was incubated with 0.8 nmol of lipid II dissolved in 4 μl of 0.5% Triton X-100. After incubation for 30 min at 37°C, the reaction was stopped by raising the temperature for 1 min to 100°C. The reaction products were separated on silica gel thin-layer plates (Silicagel 60 Merck) with the solvent chloroform/methanol/water/32% ammonia (88:48:10:1, by vol.). The products were visualized by exposure to iodine vapour. The uncleaved substrate and the reaction product 1-pyrophosphoryl-*N*-acetyl-muramyl pentapeptide-*N*-acetyl-glucosamine showed R_f_ values of 0.5 and 0.1 respectively. The areas of silica gel containing the uncleaved substrate and the product and the area in between were scraped off the plate and counted in a liquid scintillation counter. Lipid II was kindly provided by Eefjan Breukink, University of Utrecht, through Waldemar Vollmer.

### Transport of ferrichrome

Transport of [^55^Fe^3+^]ferrichrome was determined essentially as previously described (Braun *et al*., 2003). Exponentially growing cells were harvested and incubated with 1 μM [^55^Fe^3+^] (specific activity 81.5 kBq in 0.048 μg) and 5 μM deferri-ferrichrome. Samples were withdrawn after 5, 10, 15 and 25 min and filtered; the filters were dried, and the radioactivity was determined in a liquid scintillation counter.

## Supplementary material

This material is available as part of the online article from: http://www.blackwell-synergy.com/doi/abs/10.1111/

j.1365-2958.2008.06327.x

(This link will take you to the article abstract).

## References

[b1] Arié JP, Sassoon N, Betton JM (2001). Chaperone function of FkpA, a heat shock prolyl isomerase, in the periplasm of *Escherichia coli*. Mol Microbiol.

[b2] Baba T, Ara T, Hasegawa M, Takai Y, Okumura Y, Baba M (2006). Construction of *Escherichia coli* K-12 in-frame, single-gene knockout mutants: the Keio collection. Mol Syst Biol.

[b3] Behrens S, Maier R, de Cock H, Schmid FX, Gross CA (2001). The SurA periplasmic PPIase lacking its parvulin domains functions *in vivo* and has chaperone activity. EMBO J.

[b4] Benedetti H, Lloubes R, Lazdunski C, Letellier L (1992). Colicin A unfolds during its translocation in *Escherichia coli* cells and spans the whole cell envelope when its pore has formed. EMBO J.

[b5] Betton JM, Ehrmann M (2007). Periplasmic chaperones and peptidyl-prolyl isomerases. The Periplasm.

[b6] Bonhivers M, Desmadril M, Moeck GS, Boulanger P, Colomer-Pallas A, Letellier L (2001). Stability studies of FhuA, a two-domain outer membrane protein from *Escherichia coli*. Biochemistry.

[b7] Bothmann H, Plückthun A (2000). The periplasmic *Escherichia coli* peptidylprolyl cis,trans-isomerase FkpA – I. Increased functional expression of antibody fragments with and without cis-prolines. J Biol Chem.

[b8] Braun M, Endriss F, Killmann H, Braun V (2003). *In vivo* reconstitution of the FhuA transport protein of *Escherichia coli* K-12. J Bacteriol.

[b9] Braun V (1995). Energy-coupled transport and signal-transduction through the gram-negative outer-membrane via TonB-ExbB-ExbD-dependent receptor proteins. FEMS Microbiol Rev.

[b10] Braun V, Schaller K, Wabl MR (1974). Isolation, characterization, and action of colicin M. Antimicrobial Agents Chemother.

[b11] Braun V, Frenz J, Hantke K, Schaller K (1980). Penetration of colicin M into cells of *Escherichia coli*. J Bacteriol.

[b12] Braun V, Patzer SI, Hantke K (2002). Ton-dependent colicins and microcins: modular design and evolution. Biochimie.

[b13] Braun V, Braun M, Killmann H, Crosa JH, Mey AR, Payne SM (2004). Ferrichrome- and citrate-mediated iron transport. Iron Transport in Bacteria.

[b14] Buchanan SK, Lukacik P, Grizot S, Ghirlando R, Ali MM, Barnard TJ (2007). Structure of colicin I receptor bound to the R-domain of colicin Ia: implications for protein import. EMBO J.

[b15] Casadaban MJ (1976). Transposition and fusion of the *lac* genes to selected promoters in *Escherichia coli* using bacteriophage lambda and Mu. J Mol Biol.

[b16] Cascales E, Buchanan SK, Duche D, Kleanthous C, Lloubes R, Postle K (2007). Colicin biology. Microbiol Mol Biol Rev.

[b17] Chen R, Henning U (1996). A periplasmic protein (Skp) of *Escherichia coli* selectively binds a class of outer membrane proteins. Mol Microbiol.

[b18] Dreher R, Braun V, Wittmann-Liebold B (1985). Functional domains of colicin M. Arch Microbiol.

[b19] Duché D, Baty D, Chartier M, Letellier L (1994). Unfolding of colicin A during its translocation through the *Escherichia coli* envelope as demonstrated by disulfide bond engineering. J Biol Chem.

[b20] El Ghachi M, Bouhss A, Barreteau H, Touze T, Auger G, Blanot D, Mengin-Lecreulx D (2006). Colicin M exerts its bacteriolytic effect via enzymatic degradation of undecaprenyl phosphate-linked peptidoglycan precursors. J Biol Chem.

[b21] Groß P, Braun V (1996). Colicin M is inactivated during import by its immunity protein. Mol Gen Genet.

[b22] Harkness RE, Braun V (1989). Colicin M inhibits peptidoglycan biosynthesis by interfering with lipid carrier recycling. J Biol Chem.

[b23] Harkness RE, Braun V (1990). Colicin M is only bactericidal when provided from outside the cell. Mol Gen Genet.

[b24] Hoffmann H, Fischer E, Schwarz H, Braun V (1986). Overproduction of the proFhuA outer membrane receptor protein of *Escherichia coli* K-12: isolation, properties, and immunocytochemical localization at the inner side of the cytoplasmic membrane. Arch Microbiol.

[b25] Hu K, Galius V, Pervushin K (2006). Structural plasticity of peptidyl-prolyl isomerase sFkpA is a key to its chaperone function as revealed by solution NMR. Biochemistry.

[b26] Miller JH (1972). Experiments in Molecular Genetics.

[b27] Moeck GS, Tawa P, Xiang H, Ismail AA, Turnbull JL, Coulton JW (1996). Ligand-induced conformational change in the ferrichrome-iron receptor of *Escherichia coli* K-12. Mol Microbiol.

[b28] Mogensen JE, Otzen DE (2005). Interactions between folding factors and bacterial outer membrane proteins. Mol Microbiol.

[b29] Ölschläger T, Turba A, Braun V (1991). Binding of the immunity protein inactivates colicin M. Mol Microbiol.

[b30] Pilsl H, Glaser C, Groß P, Killmann H, Ölschläger T, Braun V (1993). Domains of colicin M involved in uptake and activity. Mol Gen Genet.

[b31] Postle K, Kadner RJ (2003). Touch and go: tying TonB to transport. Mol Microbiol.

[b32] Ramm K, Plückthun A (2000). The periplasmic *Escherichia coli* peptidylprolyl cis,trans-isomerase FkpA. II. Isomerase-independent chaperone activity in vitro. J Biol Chem.

[b33] Ramm K, Plückthun A (2001). High enzymatic activity and chaperone function are mechanistically related features of the dimeric *E. coli* peptidyl-prolyl-isomerase FkpA. J Mol Biol.

[b34] Rizzitello AE, Harper JR, Silhavy TJ (2001). Genetic evidence for parallel pathways of chaperone activity in the periplasm of *Escherichia coli*. J Bacteriol.

[b35] Rouviere PE, Gross CA (1996). SurA, a periplasmic protein with peptidyl-prolyl isomerase activity, participates in the assembly of outer membrane porins. Genes Dev.

[b36] Saul FA, Arie JP, Vulliez-le Normand B, Kahn R, Betton JM, Bentley GA (2004). Structural and functional studies of FkpA from *Escherichia coli*, a cis/trans peptidyl-prolyl isomerase with chaperone activity. J Mol Biol.

[b37] Schäfer U, Beck K, Müller M (1999). Skp, a molecular chaperone of gram-negative bacteria, is required for the formation of soluble periplasmic intermediates of outer membrane proteins. J Biol Chem.

[b38] Schaller K, Krauel A, Braun V (1981). Temperature-sensitive, colicin M-tolerant mutant of *Escherichia coli*. J Bacteriol.

[b39] Schaller K, Höltje J-V, Braun V (1982). Colicin M is an inhibitor of murein biosynthesis. J Bacteriol.

[b40] Schöffler H, Braun V (1989). Transport across the outer membrane of *Escherichia coli* K12 via the FhuA receptor is regulated by the TonB protein of the cytoplasmic membrane. Mol Gen Genet.

[b41] Sharma O, Yamashita E, Zhalnina MV, Zakharov SD, Datsenko KA, Wanner BL, Cramer WA (2007). Structure of the complex of the colicin E2 R-domain and its BtuB receptor. The outer membrane colicin translocon. J Biol Chem.

[b42] Sklar JG, Wu T, Kahne D, Silhavy TJ (2007). Defining the roles of the periplasmic chaperones SurA, Skp, and DegP in *Escherichia coli*. Genes Dev.

[b43] Soelaiman S, Jakes K, Wu N, Li C, Shoham M (2001). Crystal structure of colicin E3: implications for cell entry and ribosome inactivation. Mol Cell.

[b44] Studier FW, Moffatt BA (1986). Use of bacteriophage T7 RNA polymerase to direct selective high-level expression of cloned genes. J Mol Biol.

[b45] Vollmer W, Pilsl H, Hantke K, Holtje JV, Braun V (1997). Pesticin displays muramidase activity. J Bacteriol.

[b46] Wiener M, Freymann D, Ghosh P, Stroud RM (1997). Crystal structure of colicin Ia. Nature.

[b47] Zakharov SD, Cramer WA (2004). On the mechanism and pathway of colicin import across the *E. coli* outer membrane. Front Biosci.

