# Supplementary material for: Periplasmic chaperone FkpA is essential for imported colicin M toxicity
Source: Mol Microbiol. 2008 Jul 9;69(4):926–37. doi: 10.1111/j.1365-2958.2008.06327.x (PMC2615193; doi:10.1111/j.1365-2958.2008.06327.x)
Supplement: Supplementary file 1 [file mmi0069-0926-SD1.pdf]

Table S1. Specific resistance of an *fkpA* mutant to colicin M.

E. coli - Stamm	Colicin													
	A	B	D	E1	E3	IB	K	L	M	N	S4	U	5	10
JW3326 BW25113 Δ *ppiA*	+	+	+	+	+	+	+	+	+	+	+	+	+	+
JW3815 BW25113 Δ *tatC*	+	+	+	+	+	+	+	+	+	+	+	+	+	+
JW0431 BW25113 Δ *ppiD*	+	+	+	+	+	+	+	+	+	+	+	+	+	+
JW0157 BW25113 Δ *degP*	+	+	+	+	+	+	+	+	+	+	+	+	+	+
JW4103 BW25113 Δ *groL*	+	+	+	+	+	+	+	+	+	+	+	+	+	+
JW3584 BW25113 Δ *secB*	+	+	+	+	+	+	+	+	+	+	+	+	+	+
JW0427 BW25113 Δ *clpP*	+	+	+	+	+	+	+	+	+	+	+	+	+	+
JW0013 BW25113 Δ *dnaK*	+	+	+	+	+	+	+	+	+	+	+	+	+	+
JW0052 BW25113 Δ *surA*	+	+	+	+	+	+	+	+	+	+	+	+	+	+
JW3309 BW25113 Δ *fkpA*	+	+	+	+	+	+	+	+	-	+	+	+	+	+
BW25113	+	+	+	+	+	+	+	+	+	+	+	+	+	+

+, sensitive; -, insensitive. Depending on the colicin used the crude cell extracts could be diluted 10- to 1000-fold to yield a clear zone of growth inhibition on a nutrient agar plate seeded with the listed strains obtained from the Keio collection.

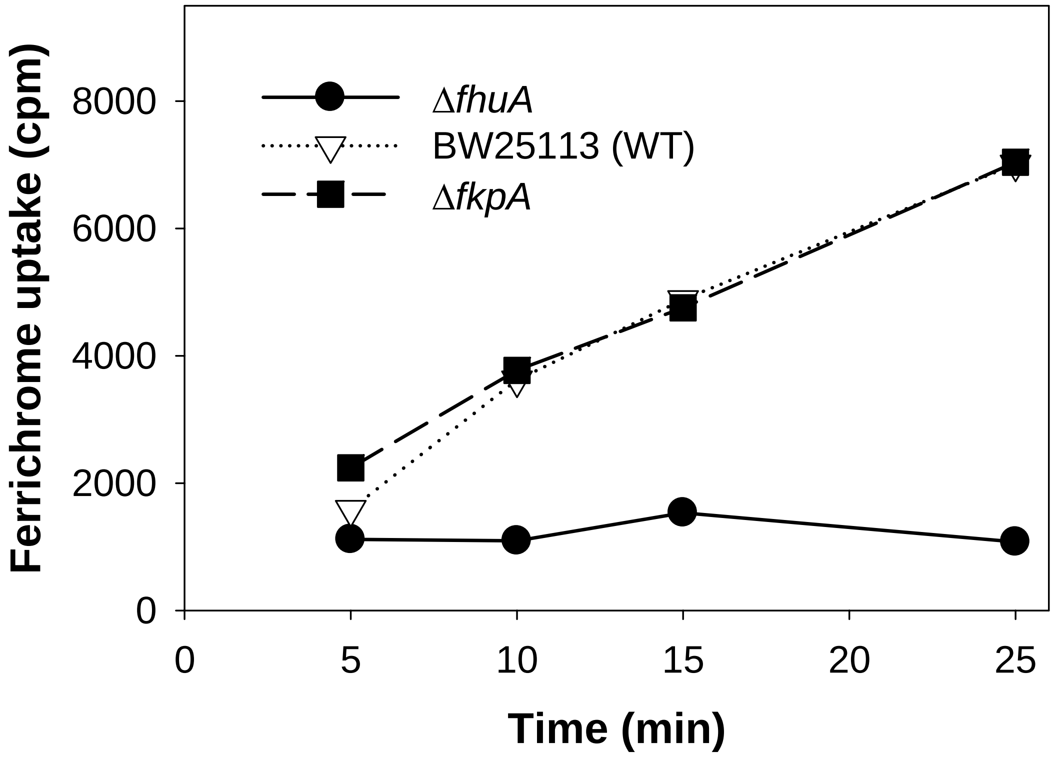

Fig. S1. Uptake of [$^{55}Fe^{3+}$]ferrichrome into cells of *E.coli* BW25113, JW3305 *fkpA*, and MB97 *fhuA*.
